# A short food frequency questionnaire to assess intake of seafood and n-3 supplements: validation with biomarkers

**DOI:** 10.1186/1475-2891-10-127

**Published:** 2011-11-19

**Authors:** Lisbeth Dahl, Camilla A Mæland, Tormod Bjørkkjær

**Affiliations:** 1 National Institute of Nutrition and Seafood Research (NIFES), PO Box 2029 Nordnes, N-5817 Bergen, Norway; 2 Montebello Center, Kurstedvegen 5, 2610 Mesnali, Norway; 3 Institute of Medicine, University of Bergen, 5021 Bergen, Norway

## Abstract

**Background:**

Seafood intake is associated with beneficial effects for human health. Seafood provides a number of nutrients beyond the traditionally known long chain marine n-3 fatty acids EPA, DPA and DHA, such as protein, vitamin D, iodine, selenium and vitamin B_12_. Valid assessment of dietary seafood and n-3 supplement intakes are becoming increasingly crucial when giving recommendations to populations as seafood consumption is regarded as an important part of a healthy and balanced diet.

**Methods:**

The aim was to validate a short FFQ developed for assessment of dietary intake of seafood and n-3 supplements using the biomarkers marine n-3 fatty acids in erythrocytes and 25(OH)D in serum.

**Results:**

Fifty-three healthy Norwegians aged 30-64 years with a mean BMI of 25 kg/m^2 ^were compliant with the study protocol. 70% reported eating seafood for dinner one to two times per week, and 45% reported to eat seafood as spread, in salads or as snack meal three to five times or more per week. The FFQ correlated significantly with both the levels of marine n-3 fatty acids (r = 0.73, p < 0.0001) and with 25(OH)D (r = 0.37, p < 0.01). Mean level of marine n-3 and of 25(OH)D were 232 ± 65 μg/g erythrocytes and 73 ± 33 nmol/L serum, respectively.

**Conclusion:**

The present short FFQ predicted strongly the levels of marine n-3 fatty acids in erythrocytes, and predicted fairly good the level of serum 25(OH)D and may therefore be a valid method for assessment of seafood and n-3 supplements intake among adults.

## Background

There is emerging interest in the potential health benefits of diets rich in seafood and long chain n-3 fatty acids (i.e. marine n-3), with evidence of a secondary protective role in coronary heart diseases [1,2]. Seafood provides several important nutrients like high-quality protein, vitamin D, vitamin B_12_, iodine and selenium besides the traditionally recognized marine n-3 fatty acids eicosapentaenoic acid (EPA, 20:5n-3), docosapentaenoic acid (DPA, 22:5n-3) and docosahexaenoic acid (DHA, 22:6n-3) [3]. Seafood, particularly fatty fish (> 5% fat) is a good source of the marine n-3 fatty acids and a portion (150 g) of salmon gives 1.5-6.0 g of marine n-3 fatty acids. In comparison, a portion (180 g) of lean fish such as cod, haddock, saithe or tusk (< 2% fat) contribute with approximately 180-350 mg marine n-3 fatty acids [4]. Other foods or supplements may also contribute with substantial amounts of marine n-3 fatty acids. For instance, the use of cod liver oil or seal oil/fish-oil capsules is common in Norway [5,6]. It should be noted that, while cod liver oil typically is rich in Vitamin D, most of the n-3 supplements from fish body oils (not liver) or concentrates are very low or devoid of vitamin D.

Biochemical measurements of specific nutrients in the blood may provide a more accurate and objective measure of dietary exposure than a food frequency questionnaire (FFQ), as it is independent of memory and knowledge of the participants [7]. For instance, EPA and DHA concentrations in blood have been used in several studies as biomarkers for assessment of dietary EPA and DHA intake measured by FFQ, as reviewed by Hodson et al. [8] and by Øverby et al. [9].

Levels of serum 25-hydroxyvitamin D (25(OH)D) are typically measured to establish the vitamin D status of an individual [10]. Vitamin D is produced in man during sun exposure, which is the main source, or provided from the diet and/or dietary supplements. It is well established that 25(OH)D varies throughout the year in response to seasonal changes in sunlight exposure, and during the winter season (October - April), the sunlight supplies ignorable amounts of vitamin D at our degree of latitude (i.e. Norway) [11,12]. Therefore, in the absence of sufficient sun exposure for dermal vitamin D synthesis, regular vitamin D intakes and/or supplements through the diet becomes important.

Valid estimates of dietary seafood intake in populations are becoming increasingly important for monitoring health effects and for dietary advisories, as regular seafood consumption is regarded an important part of a healthy and balanced diet [3,13,14]. A short-version self-administrated questionnaire with main focus on seafood and n-3 supplements was developed for the study and was based on two different previously validated FFQs assessing the habitual food intake in Norway [15-17]. The aim of the present study was therefore to validate our short-version FFQ developed for assessment of dietary intake of seafood and supplements using the biomarkers marine n-3 fatty acids in erythrocytes and 25(OH)D in serum.

## Methods

### Study population and design

Healthy volunteers working at an electrical power company (BKK) in Western Norway were invited to participate in the study. Seven hundred employees (70% men and 30% women) received the invitation by e-mail. Information about the study was also launched at the local web side of the company and through an information meeting at the work place. A total of 56 employees responded to the invitation and 53 of them (33 men and 20 women) were compliant with the study protocol. The participants received the short FFQ by e-mail three days prior to the sampling. An overnight fasting blood sample was obtained between 07.00 - 9.30 a.m. together with a morning spot sample of urine in medio April from all participants. After six months they were asked to fill in a FFQ [15,16] covering the total habitual diet, of which 47 participants returned.

### Ethics

The study protocol and all informed consent documents were approved by the Regional Committee for Medical Research Ethics in Norway and the study complies with the Declaration of Helsinki. Written informed consent was obtained from each participant before inclusion in the study and participants were free to withdraw from the study at any time without giving any reason of dismissal.

### FFQ

The short-version self-administrated questionnaire developed for the study included questions about the habitual intake of seafood for dinner, as sandwich spread, in salads or as snack meal, with questions focusing on type of seafood, frequency of intake and in some cases portion size. Frequency responses of seafood intake were recorded as: never, less than once per month, one to three times per month, one to two times per week, or three or more times per week. The portion size of seafood for dinner was recorded as follows: half a portion, one portion, one and a half portion, two portions or three portions. One portion corresponds to 150 grams of seafood; e.g. one slice of salmon fillet, three fishcakes or two deciliters of shrimps. The intake of different types of seafood was determined by asking about the intake of 36 different types of lean and fatty seafood (specific fish species, fish as sandwich spread, mollusks, crustaceans and semi-manufactured fish products). In addition it was possible to register intake of seafood not given in the list. The questionnaire also included questions about the use of supplements (cod liver oil, fish oil capsules, multivitamin/mineral mixtures, vitamin B, vitamin D, calcium and iron), in which the product names of the most commonly used supplements in Norway were listed. For the use of bottled or encapsulated cod liver oil, the questionnaire differentiated between the whole year and during winter only. Frequency response was recorded as: one to three times per month, one to three times per week, four to six times per week, or daily. The amount of intake was recorded as a teaspoon (3 mL), child's spoon (5 mL) or tablespoon (10 mL).

In addition, the short FFQ included questions concerning specific food habits, such as frequency of consumption of dairy products (a major contributor to iodine intake), fruits and vegetables, butter and margarine (products fortified with vitamin D (8 μg/100 g) in Norway) and use of fats in cooking. General characteristics like age, weight, height, smoking, physical exercise, medication and interest in eating healthy, were self reported in the questionnaire. Body mass index (BMI) was calculated as weight in kilograms divided by the square of height in meters (kg/m^2^). Frequency of exercise was determined by the question: How often do you perform physical exercise for at least 20 minutes (walking, jogging, bicycling, swimming, football, aerobics)? The questionnaire included a short written instruction about habitual intake in addition to our understanding of seafood, which comprise fish, fish products, mussels and crustaceans. On the average it took approximately ten minutes to complete the FFQ.

### Calculation of intake

**Figure 1:**
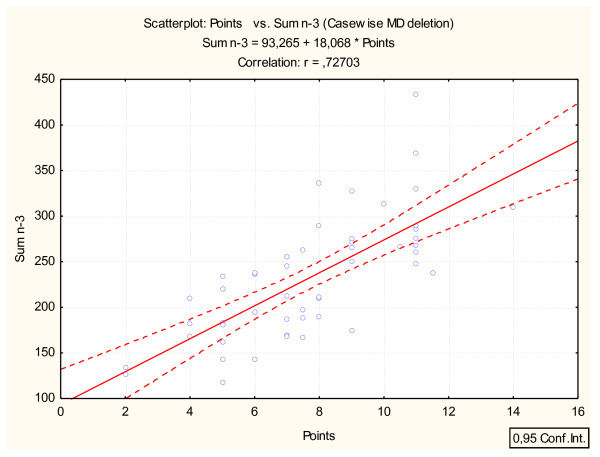
**The relationship between seafood and n-3 supplement intakes from the FFQ (points) and level of marine n-3 fatty acids in erythrocytes (μg/g)**. Points are calculated using the frequency of intake of seafood and n-3 supplements as given in Table 1.

**Figure 2:**
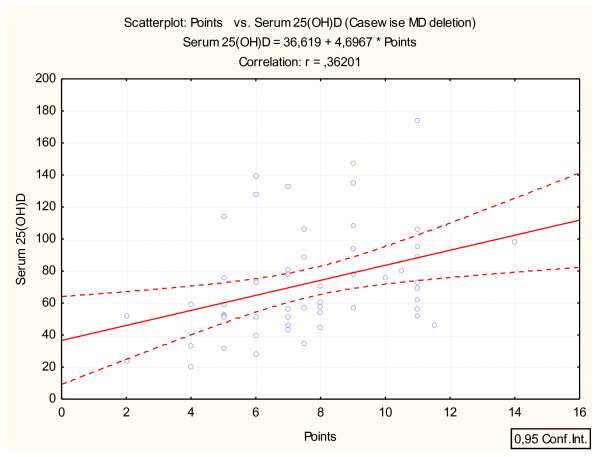
**The relationship between seafood and n-3 supplement intakes from the FFQ (points) and the level of 25-hydroxy vitamin D (25(OH)D)**. Points are calculated using the frequency of intake of seafood and n-3 supplements as given in Table 1.

In order to correlate the seafood- and n-3 supplement intakes calculated from the FFQ to fatty acid levels in erythrocytes or to 25(OH)D levels, the frequency of seafood and n-3 supplement intakes were converted into points as shown in Table 1. The points were summarized together and used in combination with the fatty acid composition in erythrocytes and the serum 25(OH)D level (Figure 1 and 2). Participants were ranked in ascending order of their number of points. They were then separated into quartiles according to frequency of seafood and n-3 supplement intakes (points). Quartile 1 (the lowest intake) equals 2-5 points, quartile 2 equals 6-7.5 points, quartile 3 equals 8-10 points and quartile 4 (the highest intake) equals 10.5-14 points.

**Table 1:** Calculation of points according to consumption frequency of seafood and omega-3 supplements.

Intake of seafood	Dinner	As spread	Intake of supplement	Liquid	Capsules
Never	0	0	Never	0	0
≤ 1 times/month	1	1	1-3 times/month	1	1
1-3 times/month	2	2	1-3 times/week	2	2
1-2 times/week	3	3	4-6 times/week	3	3
≥ 3 times/week	4	4	Daily	4	4

### Blood and urine samples

Blood samples for fatty acid composition and selenium in erythrocytes were collected in K_2 _EDTA vials, centrifuged (10 min, 1000 g, 20°C) immediately and stored at -80°C until analysis. The cell layers were adequate separated: non-hemolysed plasma clearly on the top, the buffy coat in the middle which was removed, and erythrocytes at the bottom of the tube. Fatty acid composition of total lipids in erythrocytes was determined by a simplified gas liquid chromatographic (GLC) method using 19:0 methyl esther as internal standard as previously described [18]. Some modifications were made: The methyl esters were separated using an Ultrafast Trace GC Ultra (5 min.) (Thermo Electron Corporation, Massachusetts, USA) equipped with a 5 m wax column (id: 0.1 mm, 0.2 μm film thickness; Thermo Electron Corporation), using split injection, with a temperature programme of 100°C^50°C/min^220°C ^80°C/min ^250°C and flame ionization detector. The fatty acid composition was calculated using an integrator (Chromeleon 6.80, Dionex Corporation, California, USA), connected to the GLC and identification ascertained by standard mixtures of methyl esters (Nu-Chek, Minnesota, USA). Limit of quantification (LOQ) was 10 μg fatty acid/g samples (wet weight).

Selenium and iodine were determined by inductively coupled plasma mass-spectrometry (ICP-MS) using an Agilent quadrupole ICP-MS 7500 c instrument (Yokogawa Analytical Systems Inc., Tokyo, Japan) equipped with an auto sampler ASX-500 (CETAC Technologies, Omaha, Nebraska, USA). For selenium determination, subsamples of approximately 1 g erythrocytes were submitted to microwave-assisted wet digestion using 2.0 mL concentrated nitric acid (Merck, Darmstadt, Germany) and 0.50 mL 30% w/w hydrogen peroxide (Merck, Darmstadt, Germany) in an Ethos Pro microwave system (Milestone, Sorisole, Italy). The digests were diluted to a final volume of 10 mL with deionised water (> 17 MΩ cm^-1^, Nanopure-system, Nanopure, Barnstead, UK). The accuracy was determined by use of certified reference samples i.e. Seronorm™ Trace Elements human whole blood (Sero Ltd, Asker, Norway). For iodine determination, 0.5 mL urine was diluted with 4.5 mL 1% HNO_3 _(Merck, Darmstadt, Germany) and 5 mL deionised water before analysis [19]. The trueness and the repeatability of the analytical method for iodine were established by analyzing the certified reference material Seronorm™ Trace Elements Urine (Sero Ltd, Asker, Norway).

Blood samples for 25(OH)D, lipids and thyroid hormones in serum were collected in silica gel tubes, centrifuged (10 min, 1000 g, 20°C) within two hours. Samples for 25(OH)D determination were stored frozen at -20°C until analysis. Samples for lipid and thyroid hormones were kept in the refrigerator (4°C) until analysis within 48 hours. Assays were performed at a routine clinical laboratory (Haukeland University Hospital, Bergen, Norway). Serum 25(OH)D was determined by a radioimmunoassay (RIA) method (Gamma-B 25-Hydroxy Vitamin D RIA, Immunodiagnostic System Limited (IDS), Tyne & Wear, Great Britain). The laboratory reference range of 25(OH)D was 50 to 113 nmol/L, of total cholesterol 2.9-6.1 mmol/L (age 18-29 year), 3.3-6.9 mmol/L (age 30-49 year) and 3.9-7.8 nmol/L (> 50 year), of LDL cholesterol 1.8-5.7 nmol/L, of HDL cholesterol 1.0-2.7 nmol/L (women) and 0.8-2.1 nmol/L (men), of triglycerides 0.45-2.60 mmol/L, of TSH 0.4-4.0 mIU/L, of free T_4 _10.3-24.5 pmol/L and of free T_3 _2.8-6.6 pmol/L.

### Statistical analyses

Analyses are based on participants who had completed all assessment methods (FFQ, blood and urine sample). All statistical analyses were performed using StatSoft, Inc. (2009) STATISTICA (data analysis software system), version 9.0, http://www.statsoft.com. Between group analyses were tested for normality, homogeneity of variance and subsequently analyzed with Student's t-test (if normally distributed data) or a Mann-Whitney test (if skewed data). Differences between participants were tested with paired Wilcoxon's test. Comparisons of several groups were tested with ANOVA if the data was normally distributed and with Kruskal-Wallis test if the data was skewed. The main effects were studied by a Fisher LSD test or Dunns test to identify any statistical significant differences. For all analyses a 95% confidence interval was applied where p < 0.05 was considered statistical significant.

## Results

### Baseline characteristics

Characteristics of the participants included in the validation study are given in Table 2. The BMI was significantly higher in men than in women. Fifteen men (45%) and six women (30%) were considered overweight (BMI 25.0-29.9 kg/m^2^). In addition, two men (6%) were obese (BMI 30.0-34.9 kg/m^2^). Almost 90% of the participants reported minimum 20 minutes physical exercise two or more times per week irrespective of gender. Total cholesterol (p = 0.03) and LDL cholesterol (p = 0.01) was significantly higher in men than women (Table 2).

**Table 2:** Demographic characteristics and biological measurements of the participants included in the validation study, mean ± SD.

Variables	All (n = 53)	Females (n = 20)	Males (n = 33)
Age, years	46 ± 8	44 ± 7	48 ± 9
Body mass index (BMI), kg/m^2^	25 ± 3	23 ± 3	26 ± 3
Total cholesterol, mmol/L	5.7 ± 1.0	5.3 ± 1.0	5.9 ± 1.0
LDL, mmol/L	3.9 ± 1.0	3.5 ± 1.0	4.2 ± 0.9
HDL, mmol/L	1.6 ± 0.4	1.7 ± 0.3	1.5 ± 0.4
Triglycerides, mmol/L	1.2 ± 0.6	1.1 ± 0.5	1.3 ± 0.7
TSH, mIE/L	1.97 ± 0.8	1.72 ± 0.4	2.14 ± 0.9
Free T_4_, pmol/L	18.5 ± 2.3	18.3 ± 2.4	18.7 ± 2.3
Free T_3_, pmol/L	4.6 ± 0.8	4.4 ± 0.8	4.7 ± 0.8

### Seafood intake

Table 3 shows the frequency and portion size of seafood intake of the participants. Thirty seven participants (70%) reported eating seafood for dinner one to two times per week, while 24 participants (45%) reported eating seafood as spread, in salads or as snack meal three to five times or more per week. There was no difference in frequency of seafood intake between genders, but men reported to eat significantly larger portions of seafood than women. According to the whole FFQ (n = 47) the estimated total intake of seafood was 101 ± 63 g/day. The women's (n = 18) daily intake were 88 ± 57 grams and the men's (n = 29) daily intake were 110 ± 67 grams.

**Table 3:** Frequency and portion size of intake seafood for dinner and frequency of intake of seafood as spread, in salads and as snack meal.

	All (n = 53)	Females (n = 20)	Males (n = 33)
*Seafood for dinner*			
Never	1	0	1
< 1 times/month	1	0	1
1-3 times/month	7	3	4
1-2 times/week	37	16	21
≥ 3 times more/week	7	1	6

*Portion size of dinner*			
≤ 0.5 portion	1	0	1
1 portion	26	15	11
1.5 portions	14	4	10
2 portions	11	1	10
3 portions	1	0	1

*Seafood as spread, in salads, as snack meal*			
Never	2	1	1
< 1 times/month	5	2	3
1-3 times/month	11	4	7
1-2 times/week	11	5	6
3-5 times/week	19	6	13
> 5 times/week	5	2	3

### Supplements intake

Thirty-one participants (58%) used n-3 supplements during the winter season, which was significantly more than the 25 participants (47%) that reported using these supplements during the whole year (p < 0.05). No statistical difference was found between women and men, however a tendency that women used n-3 supplements more often than men was found. Nine different n-3 supplement brands were reported and the use of capsules and liquid forms of n-3 supplements was about equal. Use of vitamin and mineral supplements was reported by 23 participants (43%).

### Fatty acid composition of erythrocytes

No significant differences were observed between men and women in the fatty acid content of erythrocytes in the present study. The mean level of saturated-, monounsaturated- and polyunsaturated fatty acids (μg/g ± SD) in erythrocytes were 891 ± 59 (39% of total fatty acids), 354 ± 47 (15% of total fatty acids) and 861 ± 88 (38% of total fatty acids), respectively. Alpha-Linolenic acid (18:3 n-3) was not detected in erythrocyte membranes, hence sum n-3 fatty acids is equal to marine n-3 fatty acids. When participants were arranged according to their increasing seafood and n-3 supplement intake, significant differences were found in the level of marine n-3 fatty acids, as shown in Table 4. Participants in quartile 4 (n = 12) had significantly higher levels of the marine n-3 fatty acids than participants in quartile 1 (n = 12, p < 0.001) and quartile 2 (n = 17, p < 0.001). In addition, participants in quartile 3 (n = 12) had significantly higher level of marine n-3 fatty acids than participants in quartile 1 (n = 12, p < 0.001). Noteworthy, five women and ten men had levels of EPA less than the limit of detection. Most of them (n = 11) were in the lowest quartile of seafood intake (Table 4).

**Table 4:** Biological measurements in blood and urine in all participants and according to seafood and n-3 supplement intake, given as mean ± SD or median.

		Seafood intake and n-3 supplement intake			
		
	All	Q1	Q2	Q3	Q4
	(n = 53)	(n = 12)	(n = 17)	(n = 12)	(n = 12)
Sum marine n-3, μg/g RBC	232 ± 65	172 ± 31^a^	206 ± 36^a^	260 ± 54^b^	298 ± 56^b^
EPA	43 ± 21	7 ± 13^a^	21 ± 16^a^	47 ± 13^b^	55 ± 26^b^
DPA	56 ± 10	48 ± 7^a^	54 ± 8^ab^	58 ± 9^b^	62 ± 11^b^
DHA	145 ± 35	117 ± 29^a^	132 ± 21^ab^	155 ± 32^b^	181 ± 27^b^
25(OH)D, nmol/L	73 ± 33	60 ± 35^a^	73 ± 35^ab^	82 ± 33^b^	83 ± 34^b^
Selenium, (μg/L)	74 ± 25	72 ± 16^a^	61 ± 16^ab^	84 ± 28^ac^	81 ± 32^ac^
Iodine, (μg/L)	102	59^a^	161^b^	78^ac^	182^bc^

Different letters denote significant difference between quartile groups in the same row.

### Correlation between short FFQ and levels of marine n-3 fatty acids or 25(OH)

Seafood and n-3 supplement intakes calculated by the short FFQ were found to correlate significantly with the level of marine n-3 in erythrocytes (r = 0.73, p < 0.0001) (Figure 1) and with the serum level of 25(OH)D (r = 0.37, p < 0.01) (Figure 2). The correlation of erythrocyte EPA, DPA and DHA with the short FFQ was 0.69, 0.66 and 0.52, respectively (p < 0.001). Mean level of marine n-3 fatty acids was 253 μg/g in erythrocytes of users of n-3 supplements (n = 31) which is significantly higher than for non-users of n-3 supplements (n = 22, 201 μg/g p < 0.01). Users of n-3 supplements (n = 31) had significantly higher level of 25(OH)D than non-users (n = 22) (73 nmol/L in users and 53 nmol/L in non-users, p < 0.05). Users of other types of supplements than n-3 containing vitamin D (n = 11) also showed a significant higher level of 25(OH)D than non-users (n = 42) (98 nmol/L in users and 58 nmol/L in non-users, p < 0.05). Users of margarine (n = 26) had a median level of 25(OH)D of 75 nmol/l compared with non-users of margarine (n = 27) who had 58 nmol/L (p > 0.05).

Classification into quartiles of seafood and n-3 supplements intakes in relation to erythrocytes level of marine n-3 fatty acids are given in Table 5. This analysis correctly assigned 28 participants (53%) into the same quartile and only two participants (4%) were grossly misclassified (i.e. classified into opposing quartile), showing that 96% were correctly classified into same or adjacent quartile (Table 5). For the classification of seafood and n-3 supplement intakes and level of 25(OH)D, only 16 participants (30%) were correctly classified into the same quartile; 41 participants (77%) were correctly classified into the same or adjacent quartile and twelve (23%) were grossly misclassified (Table 6).

**Table 5:** Agreement of quartile assignment between the short FFQ and n-3 concentration in erythrocytes.

Quartile	Same quartile	Adjacent quartile	Misclassified
1 (n = 12)	5	5 (above)	2 (above)
2 (n = 17)	8	9 (4 above, 5 below)	0
3 (n = 12)	7	5 (below)	0
4 (n = 12)	8	4 (below)	0
Total number (n = 53)	28	23	2
%	53	43	4

**Table 6:** Agreement of quartile assignment between the short FFQ and vitamin D, measured as 25(OH)D in serum.

Quartile	Same quartile	Adjacent quartile	Misclassified
1 (n = 12)	5	5 (above)	2 (above)
2 (n = 17)	4	9 (4 above, 5 below)	4 (above)
3 (n = 12)	3	7 (4 above, 2 below)	1 (below)
4 (n = 12)	4	4 (below)	5 (below)
Total number (n = 53)	16	25	12
%	30	47	23

The correlation between marine n-3 fatty acid in erythrocytes and the FFQ covering the whole diet (filled in six months after the short FFQ) was 0.67 (p < 0.001). The correlation between the two FFQs (i.e. short FFQ and whole diet FFQ) was 0.59 (p < 0.001). The correlation of erythrocyte EPA, DPA and DHA with the whole FFQ was 0.73, 0.50 and 0.69, respectively. For 25(OH)D and the whole diet FFQ the correlation was 0.52 (p < 0.001). When supplements were excluded the correlation was 0.41 between 25(OH)D and the whole diet FFQ.

### Levels of Vitamin D and selenium in blood and iodine in urine

Forty two (79%) participants had serum 25(OH) D concentrations considered optimal (≥ 50 nmol/L), nine participants (17%) had levels considered suboptimal (25-49 nmol/L) and two participants (4%) were considered deficient (< 12.5-24 nmol/L). There was no significant differences in 25(OH)D status between gender, but participants in quartile 1 (n = 12) had significantly lower level of 25(OH)D than participants in quartile 3 (n = 12) and quartile 4 (n = 12) (p < 0.01) (Table 4). A tendency that the level of 25(OH) increased with increasing age was observed, however this trend was not significant.

Selenium concentration in plasma varied from 33 to 170 μg/L with mean ± SD level in men and women of 72 ± 33 and 74 ± 17 μg/L, respectively. Participants in quartile 2 (n = 17) had significantly lower level of selenium than participants in quartile 3 (n = 12) and 4 (n = 12) (p < 0.05). (Table 4).

The range of urinary iodine concentration was wide (27-632 μg/L) with median level of 102 μg/L, however the iodine excretion in men was significantly higher (134 μg/L) in comparison with women (84 μg/L). Importantly, all participants had values of TSH, T_3 _and T_4 _within the laboratory's reference range, indicating that all of them had adequate iodine nutrition.

## Discussion

In nutrition research including clinical settings, a short FFQ could be a powerful tool for estimating dietary exposures of interest. By using the present short FFQ focusing on seafood and n-3 supplements, we found a strong correlation (r = 0.73) with the level of marine n-3 fatty acids in erythrocytes. This correlation was weaker when the intake of n-3 supplements was not included in the analysis (r = 0.61), however still strong enough in view of expected correlations when validating a FFQ against a biomarker in blood matrices [9]. Other validation studies of different FFQs against total n-3 fatty acids, EPA or DHA in erythrocytes, plasma or serum reported correlation coefficients in the range of 0.35-0.67 [7,15,20,21]. Similar correlations are reported when weighed records are validated against serum or plasma levels of marine n-3 fatty acids, as reviewed by Øverby et al [9]. The lower correlation between the seafood FFQ and marine n-3 fatty acids in erythrocytes when the supplements were excluded illustrate the importance of including n-3 supplement registration in the FFQ. This was further emphasized by the finding that the users of n-3 supplements had significantly higher levels of marine n-3 in erythrocytes than non-users. The importance of including questions about supplements intakes are in agreement with other studies focusing on dietary assessment methods [22,23]. At the same time, it is of importance to include seafood in the diet as it provides several important nutrients beyond the marine n-3 fatty acids. Further, increased seafood consumption is in accordance with recommendations of a healthy and balanced diet [3].

Very few validation studies are designed to compare dietary vitamin D intake with the biomarker 25(OH)D [23]. Although no information on sunlight exposure was collected in the present study, we found a fairly good correlation between the FFQ and serum 25(OH)D. This might be explained by the fact that the present study was conducted in late spring (i.e. April), however adding questions regarding residence, ethnicity and sun exposure (e.g. sun beds, sun seeking holidays, time spent in daylight) are essential in the later revision of the present short FFQ. In absence of sufficient sunlight exposure, dietary vitamin D intakes become important [10]. Further, the seasonal differences in the correlation between vitamin D intake and 25(OH)D are described in several studies. The MoBa study [22] showed a stronger correlation between vitamin D intake calculated by FFQ or by food record and plasma 25(OH)D concentration (0.45 and 0.51, respectively) during the winter months in comparison with the whole year (0.32 and 0.43). In the study by Youl and Hee-Kyung [24] frequent fish intake and regular exercise were significantly associated with higher level of 25(OH)D in healthy Korean men. Fish is regarded as one of the major sources of vitamin D in the Korean diet and their study was also conducted during a time period in which solar UV radiation was very low [24]. A different aspect with the present study is that even if mean level of vitamin D was in the range of optimal status, still one fifth of the participants had suboptimal vitamin D-status (25(OH)D < 50 nmol/L), despite the fact that our study included healthy adults reporting to be apparently interested in diet and health. However, this underlines the growing global health concern about vitamin D insufficiency, particularly in Nordic countries during the winter season, and the present study mirrors the results of Holvik et al. [25].

Most studies use national food composition data when converting intakes of different foods into nutrient intakes. However, in the present short FFQ the frequency of seafood consumption and supplements intakes were converted into points rather than into quantities of intake, partly because the portion size of intake was not reported in all questions and partly because no Norwegian food composition table is currently publicly available for individual values of fatty acids. The Norwegian food composition table gives only the sum of saturated, monounsaturated or polyunsaturated fat in the foods [26]. Nevertheless, it is important to consider that these food databases often includes analyses of nutrients from a limited number of foods, although these foods should be representative of the given food group. Further, the specific nutrient content in foods may vary within the same food group due to different brands and due to seasonal variations [7,21].

A weakness of the calculation of points is that the same total number of points might reflect a very different amount of seafood intake, e.g. eating seafood for dinner three times or more per week gives four points (Table 1). In comparison, you will also get a total of four points by eating seafood for dinner once a month plus eating seafood as spread one to two times per week. If you have seafood for dinner three times per week, the seafood intake is about 360-450 grams assuming a portion size of 120-150 grams. This intake is considerably higher compared to seafood for dinner once per month (portion size of 120-150 g) together with seafood as spread one to two times per week (portion size of 25-50 g), where the total seafood intake will be in the range of 53-135 g (assuming that the dinner contributes with approximately 28-35 grams plus the intake from spread of 25-100 grams). Even so, the short FFQ was able to adequately identify participants according to their erythrocyte level of marine n-3 fatty acids. The degree of strongly misclassification data meaning being classified into opposite quartile was very small, and 96% of the participants were classified into the same quartile or adjacent quartile by the short FFQ and by the marine n-3 fatty acids. This was similar to that reported in other studies [27-30]. Further, an important finding of the present study was that the correlation between seafood intake using the FFQ covering the whole diet and marine n-3 fatty acids in erythrocytes (0.67) was similar to what we found when we used the short FFQ (0.73). This also applies to the correlation with both FFQs and vitamin D.

Today there is no method available to measure dietary exposure among individuals or groups of individuals without error. Therefore, it is important that a new FFQ is tested for reproducibility and validity [31]. In the present study we have only used an independent validity check of intakes as we have used biomarkers and we have not tested the relative validity by assessing the short FFQ to a reference method (i.e. weighted food records). The advantage of using a biomarker as a reference method is that the errors of the biomarker method are different from the errors of a dietary method. Further, we did not test the reproducibility of the short FFQ. However, the questions in the short FFQ are based on two different Norwegian FFQs covering the whole diet which both have been validated [16,17]. Since we found a significant correlation between the short FFQ and the whole FFQ (the whole FFQ was filled in six months after the short FFQ), we believe that this strengthen our validation, although this design cannot fully replace a measure of reproducibility.

The marine n-3 fatty acids in adipose tissue or in blood are the most frequently used biomarkers for intake of seafood [8]. Interestingly, we found a larger correlation between the short FFQ and the DPA level than reported in several other studies [28,29]. The content of DPA in seafood varies in accordance to the fattiness of the species, and the content of DPA in seafood is normally lower than the level of EPA and DHA [4]. Although, the intake of DPA is lower compared with EPA and DHA, the DPA level in erythrocytes is higher than the EPA level. We can't give a definite explanation, however we suggest that this is due to some methodological issues. The method for determination of fatty acids in some studies [21,26,28] find low correlation for DPA compared to our study and some other studies using a different method for the determination of fatty acids [16,17].

In a study by Brantsæter et al [32], the authors explore several biomarkers for total fish intake and suggest that blood arsenic concentration appears to be a potential biomarker for fish and seafood intake. Arsenic intake, mainly in the form of arsenobetaine, is largely determined by the amount of seafood consumed in areas without arsenic-contaminated drinking water exposure [33]. Examination of results in the present study revealed no correlation between blood arsenic concentration and the short FFQ (data not shown). However, in our study the arsenic concentration was determined in erythrocytes and not in whole blood [32]. On the contrary, our study confirms the finding that urinary iodine excretion (μg/L) is not suitable as biomarker for seafood intake [32]. This is shown in our study as a higher urinary iodine excretion of participants in quartile 2 than in quartile 3 (Table 4) and can be explained by the fact that milk and dairy products are consumed more regularly (i.e. every day) than seafood (i.e. weekly). Important iodine sources in the Norwegian diet are seafood, milk and dairy products and eggs [34]. Worth mentioning is the tendency that the participants with urinary iodine concentration less than 100 μg/L reported lower consumption of dairy products and seafood than participants with urinary iodine concentration above 100 μg/L. Another important aspect is that we have used median iodine concentration from spot samples. To avoid misinterpretation of the data on the iodine status due to variation in the urinary volume, use of estimated 24-hour urinary iodine excretion adjusting for age and gender specific creatinine excretion has been suggested [35].

The present validation study was conducted among middle-aged adults with a higher consumption of seafood compared to national dietary studies performed previously in Norway [36,37]. While convenient for feasibility (e.g. recruitment), whether the present study population was fully representative of the Norwegian adult population in terms of socio-demographic variables remains unanswered. When recruiting volunteers for the study we announced for participants to participate in a seafood study. This might have resulted in that non-seafood consumers avoided signing up for the study, as only one participant reported no seafood intake.

## Conclusion

The present short FFQ provides useful and valid information on seafood and supplement intakes among adults. We showed that the short FFQ may strongly predict levels of marine n-3 fatty acids in erythrocytes, and fairly good predicts level of serum 25(OH)D. Valid assessment of dietary seafood and supplement intake is becoming increasingly crucial when giving recommendations in a population, as seafood consumption is regarded as an important part of a healthy and balanced diet.

## Abbreviations

EPA: eicosapentaenoic acid, 20:5(n-3); DPA: docosapentaenoic acid, 22:5(n-3); DHA: docosahexaenoic acid, 22:6(n-3); FFQ: food frequency questionnaire; 25(OH)D: 25-hydroxy Vitamin D; BMI: body mass index (kg/m^2^); LOQ: limit of quantification; GLC: gas liquid chromatographic; ICP-MS: inductively coupled plasma mass-spectrometry.

## Competing interests

The authors declare that they have no competing interests.

## Authors' contributions

LD, CAM and TB have made equally contributions to design, collection of data, analysis and interpretation of data in the study. LD has led manuscript writing and all authors have been involved in critically drafting the manuscript. All authors read and approved the final version of the manuscript.
